# Ψ-Atlas: An Integrated Atlas for Pseudouridine Epitranscriptome

**DOI:** 10.1093/gpbjnl/qzag004

**Published:** 2026-01-23

**Authors:** Xiaochen Wang, Jinjing Luo, Xiaoqiang Lang, Yongqing Ling, Yiming Zhou, Guoxian Liu, Xiangye Chen, Yibo Chen, Yingshun Zhou, Yi Cao, Zhonghui Zhang, Changjun Ding, Demeng Chen, Qi Liu

**Affiliations:** Rice Research Institute, Guangdong Academy of Agricultural Sciences, Guangdong Key Laboratory of Rice Science and Technology, Key Laboratory of Genetics and Breeding of High Quality Rice in Southern China (Co-construction by Ministry and Province), Ministry of Agriculture and Rural Affairs, Guangdong Rice Engineering Laboratory, Guangzhou 510640, China; Center for Translational Medicine, The First Affiliated Hospital, Sun Yat-sen University, Guangzhou 510080, China; State Key Laboratory of Tree Genetics and Breeding, Key Laboratory of Tree Breeding and Cultivation of State Forestry Administration, Research Institute of Forestry, Chinese Academy of Forestry, Beijing 100091, China; Department of Pathogenic Biology, School of Basic Medical, Southwest Medical University, Luzhou 646000, China; State Key Laboratory of Tree Genetics and Breeding, Key Laboratory of Tree Breeding and Cultivation of State Forestry Administration, Research Institute of Forestry, Chinese Academy of Forestry, Beijing 100091, China; Microbiology and Metabolic Engineering Key Laboratory of Sichuan Province, College of Life Science, Sichuan University, Chengdu 610041, China; Rice Research Institute, Guangdong Academy of Agricultural Sciences, Guangdong Key Laboratory of Rice Science and Technology, Key Laboratory of Genetics and Breeding of High Quality Rice in Southern China (Co-construction by Ministry and Province), Ministry of Agriculture and Rural Affairs, Guangdong Rice Engineering Laboratory, Guangzhou 510640, China; Guangdong Provincial Key Laboratory of Biotechnology for Plant Development, School of Life Science, South China Normal University, Guangzhou 510631, China; Rice Research Institute, Guangdong Academy of Agricultural Sciences, Guangdong Key Laboratory of Rice Science and Technology, Key Laboratory of Genetics and Breeding of High Quality Rice in Southern China (Co-construction by Ministry and Province), Ministry of Agriculture and Rural Affairs, Guangdong Rice Engineering Laboratory, Guangzhou 510640, China; Guangdong Provincial Key Laboratory of Biotechnology for Plant Development, School of Life Science, South China Normal University, Guangzhou 510631, China; Rice Research Institute, Guangdong Academy of Agricultural Sciences, Guangdong Key Laboratory of Rice Science and Technology, Key Laboratory of Genetics and Breeding of High Quality Rice in Southern China (Co-construction by Ministry and Province), Ministry of Agriculture and Rural Affairs, Guangdong Rice Engineering Laboratory, Guangzhou 510640, China; College of Life Science and Technology of Huazhong Agricultural University, Wuhan 430000, China; Center for Translational Medicine, The First Affiliated Hospital, Sun Yat-sen University, Guangzhou 510080, China; Rice Research Institute, Guangdong Academy of Agricultural Sciences, Guangdong Key Laboratory of Rice Science and Technology, Key Laboratory of Genetics and Breeding of High Quality Rice in Southern China (Co-construction by Ministry and Province), Ministry of Agriculture and Rural Affairs, Guangdong Rice Engineering Laboratory, Guangzhou 510640, China; Department of Pathogenic Biology, School of Basic Medical, Southwest Medical University, Luzhou 646000, China; Microbiology and Metabolic Engineering Key Laboratory of Sichuan Province, College of Life Science, Sichuan University, Chengdu 610041, China; Guangdong Provincial Key Laboratory of Biotechnology for Plant Development, School of Life Science, South China Normal University, Guangzhou 510631, China; State Key Laboratory of Tree Genetics and Breeding, Key Laboratory of Tree Breeding and Cultivation of State Forestry Administration, Research Institute of Forestry, Chinese Academy of Forestry, Beijing 100091, China; Center for Translational Medicine, The First Affiliated Hospital, Sun Yat-sen University, Guangzhou 510080, China; Rice Research Institute, Guangdong Academy of Agricultural Sciences, Guangdong Key Laboratory of Rice Science and Technology, Key Laboratory of Genetics and Breeding of High Quality Rice in Southern China (Co-construction by Ministry and Province), Ministry of Agriculture and Rural Affairs, Guangdong Rice Engineering Laboratory, Guangzhou 510640, China

**Keywords:** Pseudouridine, Next-generation sequencing, Direct RNA sequencing, PsiVar, PsiFinder

## Abstract

Pseudouridine (Ψ) is a C^5^ glycosidic isomer of uridine, formed by breaking the N^1^ glycosyl bond and undergoing a 180° base rotation. This modification is one of the most widespread post-transcriptional alterations in RNA and is universally distributed among diverse RNA species. The pervasiveness of this modification enhances RNA structural integrity, confers unique structural and functional attributes upon the RNA molecules it adorns, and facilitates additional hydrogen bonding. However, a convenient, integrated, and intuitive visualization database that includes all currently reported species and RNA types is lacking. Here, we present Ψ-Atlas, an extensive database meticulously curated for the comprehensive collection and annotation of RNA pseudouridine. This database encompasses 554,895 Ψ modification sites across various RNA categories, including mRNA, non-coding RNA (ncRNA), tRNA, and rRNA, in 77 distinct species reported in the current literature. The sequencing methodologies employed comprise next-generation sequencing techniques such as Ψ-Seq, Pseudo-Seq, CeU-Seq, PSI-Seq, RBS-Seq, HydraPsiSeq, BID-Seq, and PRAISE-Seq, as well as third-generation sequencing methods like direct RNA sequencing. Ψ-Atlas is the most comprehensive and integrated resource for RNA Ψ modifications to date. Ψ-Atlas offers an intuitive interface for information display and a myriad of analytical tools, including PsiVar and PsiFinder. Overall, this platform serves as a robust search and visualization tool for the study of pseudouridylation in epitranscriptomics. Ψ-Atlas is available at https://rnainformatics.org.cn/PsiAtlas.

## Graphical abstract

**Figure:**
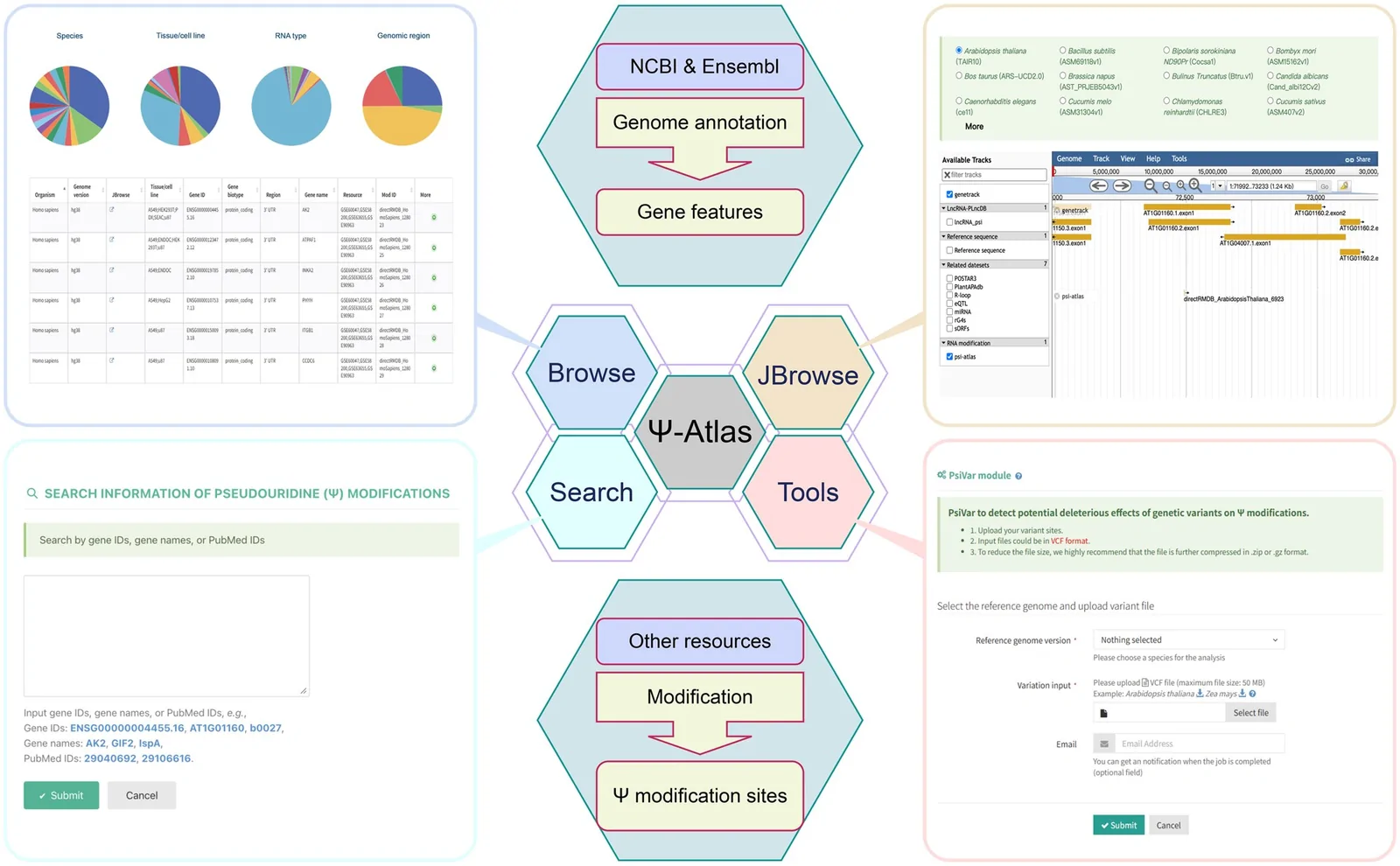

## Introduction

Currently, over 170 distinct ribonucleotide modifications occurring in RNA have been identified [1,2]. Among these, pseudouridine (Ψ), an isomer of uridine (U) at the C^5^-glycoside, is one of the earliest identified and most abundant ribonucleotide modifications [3]. It is ubiquitously present in both coding RNAs and various non-coding RNAs (ncRNAs), including rRNA, tRNA, long non-coding RNA (lncRNA), small nuclear RNA (snRNA), and small nucleolar RNA (snoRNA) [3–6]. Many Ψ positions in rRNA are highly conserved. For instance, *Escherichia coli* 23S rRNA includes three conserved Ψ residues at positions 1911, 1915, and 1917 [6,7]. Mitochondrial rRNA is encoded by the mitochondrial genome and is subject to pseudouridylation. One identified modification site is Ψ1397 in *Homo sapiens* mitochondrial LSU 16S rRNA, located near the peptidyl transferase center (PTC) core [8]. In most tRNAs, Ψ is found not only in the conserved TΨC loop but also in the D stem and/or anticodon stem and loop [9]. Additionally, Ψ is conserved in spliceosomal snRNAs, such as U1, U2, U4, U5, and U6. Its specific locations are primarily within regions crucial for RNA–RNA and RNA–protein interactions essential for spliceosome assembly and splicing processes [10–12]. In the early stages of Ψ research, ncRNA was the primary focus. However, with the advancement of high-throughput technologies, an increasing number of Ψ sites have been discovered in mRNA in *Saccharomyces cerevisiae* and *H. sapiens* [13–15].

Early investigations into the structure of Ψ residues have, to a certain extent, laid the groundwork for later elucidating the functional implications of pseudouridylation in RNA. Due to the presence of two hydrogen donors (N^1^ and N^3^ imino protons) and two hydrogen acceptors in Ψ [16], when Ψ pairs with A, G, U, or C within the double helix to form Ψ-A, Ψ-G, Ψ-U, or Ψ-C pairs, it enhances the thermodynamic stability of RNA duplexes. This results in a more stable base stacking arrangement and a more rigid RNA structure compared to U [16–18]. These structures and their thermodynamic properties contribute to the specific functions elicited by Ψ *in vivo*. For instance, pseudouridylation of rRNA optimizes the function of distinct ribosomal subpopulations, enabling the translation process and cellular proteome to adapt to specific intracellular or environmental conditions [19]. Depletion of the *RluD* gene in *E. coli* results in the absence of Ψ at positions 1911, 1915, and 1917 in the 23S rRNA, leading to a significant inhibition of *E. coli* growth [20]. Depletion of the essential enzyme RNA Pseudouridine Synthase D4 (RPUSD4), responsible for the Ψ1397 modification in *H. sapiens* mitochondrial LSU 16S rRNA, reduces the activity of electron transport chain complexes, thereby impeding mitochondrial translation [8]. In *Arabidopsis thaliana*, Leaf Curly and Small 1 (FCS1), located in the mitochondria, has been identified as a mediator of pseudouridylation in mitochondrial 26S rRNA. In *fcs1* mutants, although mitochondrial gene transcripts increase, protein accumulation is reduced. This results in severe impairment of mitochondrial structure and function, consequently affecting the growth and development of *A. thaliana* [4]. In tRNA, Ψ stabilizes its secondary structure, influencing the rate and accuracy of translation, thereby playing a pivotal role in directing translational control in stem cells [21]. Ψ residues in snRNA aggregate in crucial functional regions, actively participating in precursor mRNA splicing. On spliceosomal U6 snRNA, the Ψ residue at position 28 (Ψ28), catalyzed by Pseudouridine Synthase 1p (Pus1p) during *S. cerevisiae* filamentous growth, contributes to this process [22]. Pseudouridylation contributes to the development of antiviral mRNA vaccines by reducing mRNA immunogenicity and attenuating innate immune recognition [23–26].

*In vivo* synthesis of Ψ is generally mediated by enzymes known as pseudouridine synthases (PUSs). Within this synthase superfamily, members of the tRNA pseudouridine(38–40) synthase (TruA), tRNA pseudouridine(55) synthase (TruB), and tRNA pseudouridine(13) synthase (TruD) families are involved in tRNA modification, whereas members of the bifunctional tRNA pseudouridine(32) synthase/23S rRNA pseudouridine(746) synthase (RluA) and 16S rRNA pseudouridine(516) synthase (RsuA) families primarily participate in Ψ modifications of rRNA [27]. This synthesis mechanism is universally present in diverse organisms, including eukaryotes, prokaryotes, and archaea [28]. For instance, TruB (bacterial origin) and Pus10 (archaeal origin) have demonstrated the ability to modify U at position 55 of tRNA to Ψ [27,29–31]. Another mechanism for Ψ formation involves H/ACA box ribonucleoproteins (RNPs) containing the ANANNA or ACA motifs, which isomerize specific Us in rRNA, U2 snRNA, and mRNA into Ψ [3,32].

In addition to the early identification of Ψ using mass spectrometry (MS) [33], techniques for detecting Ψ have continually evolved with the advancement of sequencing technologies. Specifically, chemical sequencing methods based on next-generation sequencing (NGS) technologies, such as Ψ-Seq, Pseudo-Seq, PSI-Seq, and CeU-Seq, have been developed [34–37]. Recently, with the introduction of direct RNA sequencing (DRS), Ψ mapping has reached a new pinnacle [38]. Therefore, a comprehensive Ψ map database covering all species and RNA types is crucial. However, existing comprehensive databases, such as RMDisease (v2.0) [39], DirectRMDB [38], RMBase (v3.0) [40], and PRMD [41], contain only a small number of Ψ modification sites. Moreover, to date, there is no dedicated Ψ modification database that spans multiple species and integrates data from diverse technologies. To this end, we established the Ψ-Atlas website, encompassing 77 species and 554,895 Ψ modification sites (https://rnainformatics.org.cn/PsiAtlas). This platform serves as a robust search and visualization tool for the study of pseudouridylation in epitranscriptomics.

## Database implementation

The Ψ-Atlas platform was developed using HTML, PHP, CSS, and JavaScript, and is hosted in an Apache environment on a Linux system equipped with four Octa-core AMD processors (each clocked at 2.6 GHz) and 512 GB of RAM. All collected and processed datasets, together with their associated metadata, are stored and managed in a MySQL database. The back-end data-processing workflows were implemented using a combination of Python, Perl, and R, among which R packages including ggplot2, cowplot, and ggpubr were employed to generate bitmap figures in PNG and PDF formats. For the front-end, JavaScript libraries such as jQuery, DataTables, and Highcharts were embedded to enable dynamic and interactive data exploration and querying, and JBrowse [42] was integrated as a web-based genome browser to display the distribution of Ψ modification sites at the genomic scale.

## Database content and usage

### Data collection and content of Ψ-Atlas

**Figure 1:**
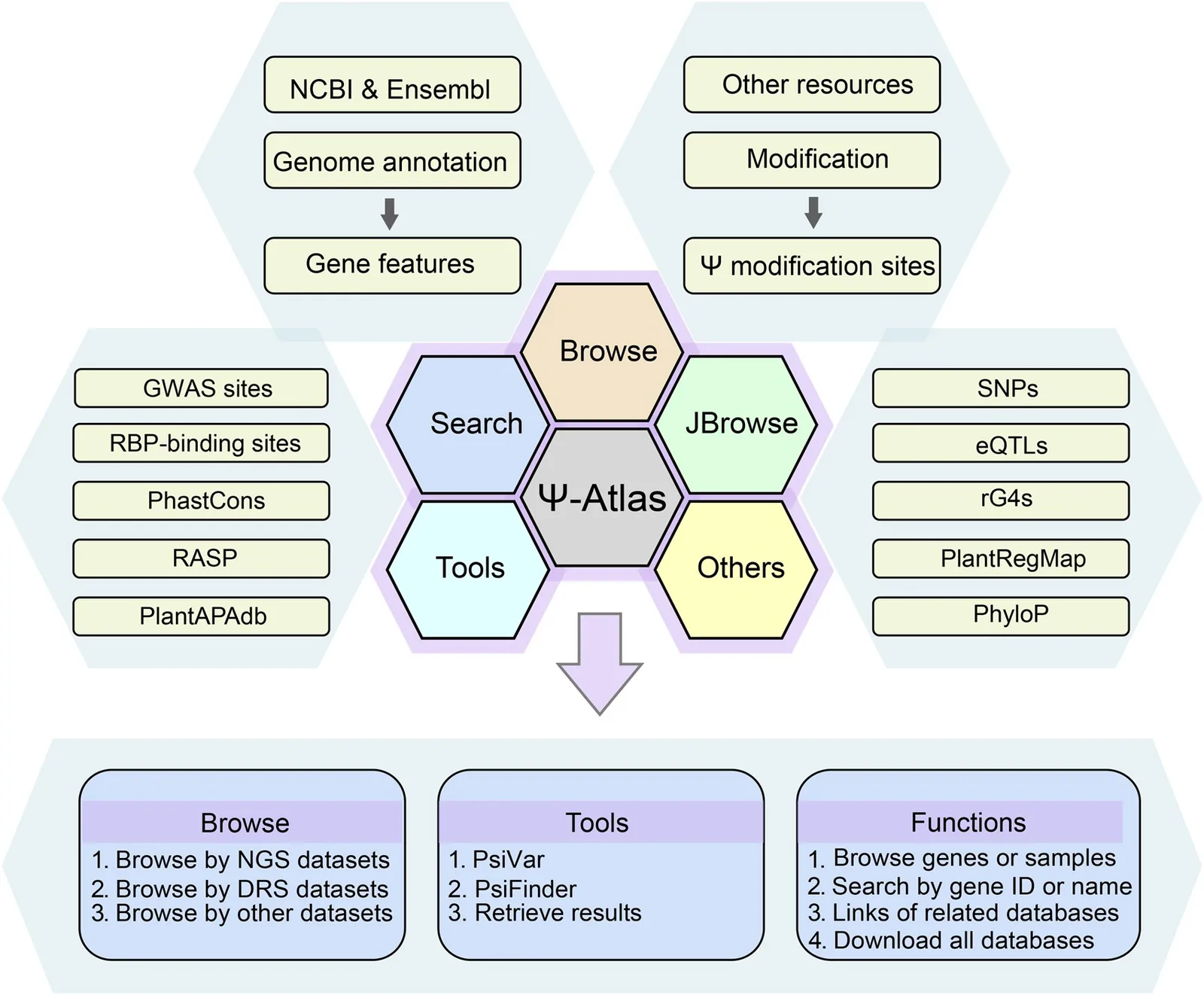
Overall workflow of Ψ-Atlas Ψ-Atlas is a comprehensive and extensive database that systematically collects and annotates Ψ modification sites. The database contains 554,895 Ψ modification sites reported in the current literature from 77 different species, including mRNA, ncRNA, tRNA, rRNA, and other RNA categories. Ψ-Atlas offers an intuitive interface for information display and two analytical tools, PsiVar and PsiFinder. Ψ, pseudouridine; ncRNA, non-coding RNA; NCBI, National Center for Biotechnology Information; GWAS, genome-wide association study; RBP, RNA-binding protein; RASP, RNA Atlas of Structure Probing; SNP, single-nucleotide polymorphism; eQTL, expression quantitative trait locus; rG4, RNA G-quadruplex; NGS, next-generation sequencing; DRS, direct RNA sequencing.

**Figure 2:**
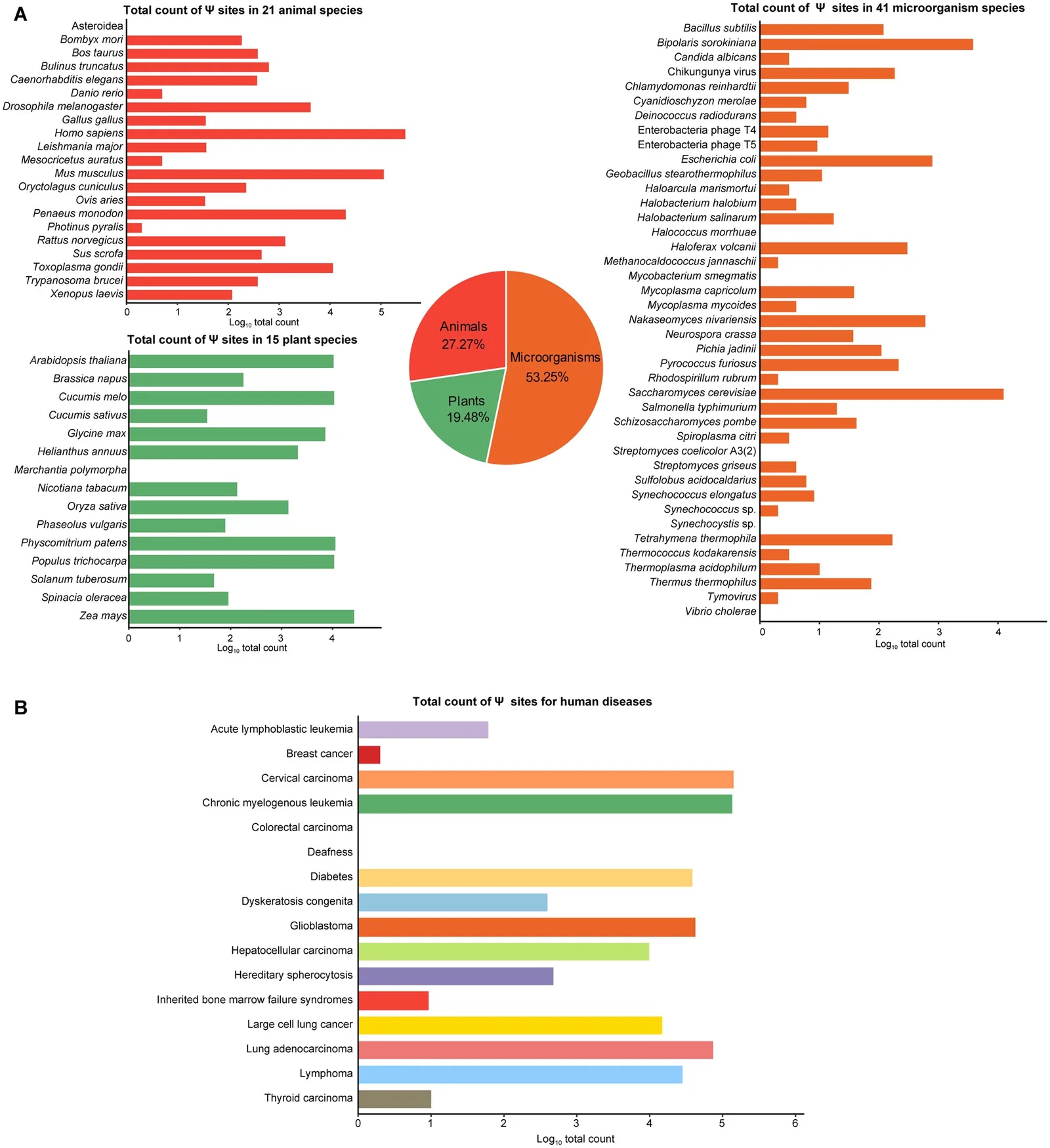
Ψ sites in Ψ-Atlas across species and diseases **A**. Statistics of the Ψ sites for each species. **B**. Statistics of Ψ sites for human diseases.

We developed Ψ-Atlas, the most comprehensive and integrated resource for RNA Ψ modifications to date (**Figure 1**). The database spans 77 species, comprising 21 animals, 15 plants, and 41 microorganisms, and currently contains 554,895 Ψ sites, among which *H. sapiens* (301,335), *Mus musculus* (113,961), *Penaeus monodon* (20,364), *A. thaliana* (10,509), *Cucumis melo* (10,683), and *S. cerevisiae* (12,270) are the most extensively profiled species (**Figure 2**A; Table S1). In addition, Ψ-Atlas records the number of Ψ modifications, associated RNA types, and related information across 16 human disease types, including acute lymphoblastic leukemia, cervical carcinoma, diabetes, and dyskeratosis congenita (Figure 2B). All of these datasets were processed through a standardized pipeline, stored in a MySQL database, and presented in a user-friendly web module, and are available through the “Download” page. We further developed interactive analytical tools, including PsiFinder for identifying putative Ψ sites in genomic regions of interest and PsiVar for detecting the potential effects of genetic variants on Ψ modification sites.

In total, we collected and integrated all currently published Ψ modification data from 132 studies, encompassing existing databases, high-throughput sequencing datasets, and results validated by non-high-throughput experiments; the accession numbers of all raw data sources are provided in Table S2. The detection methods represented in Ψ-Atlas fall into three categories. The first comprises MS- or chromatography-based techniques, such as liquid chromatography–mass spectrometry (LC-MS) [33,43–45], SILNAS [46], pseudo-MS^3^ [47], and two-dimensional thin-layer chromatography (2D-TLC) [48]. The second comprises chemical- or antibody-based approaches coupled with NGS technologies, including PRAISE-Seq [49], Ψ-Seq [50], Pseudo-Seq [36], CeU-Seq [34], PSI-Seq [37], small RNA Ψ-Seq [51], RBS-Seq [52], HydraPsiSeq [14], BID-Seq [53], CLAP (not coupled with high-throughput sequencing) [54], and PA-Ψ-Seq [55]. The third comprises nanopore-based “third-generation” sequencing technologies, including nanoRMS [56], NanoPsu [57], and DRS [38]. Ψ sites with precise genomic coordinates were derived mainly from the chemical-plus-NGS and nanopore-based methods, whereas sites validated by wet-lab experiments but lacking genomic coordinates were integrated into a unified dataset under the “Browse of other datasets” section of Ψ-Atlas.

The reference genome files and their corresponding annotation files were downloaded from GENCODE [58] for human and mouse, and from Ensembl and National Center for Biotechnology Information (NCBI) for the remaining species. LiftOver (https://genome-store.ucsc.edu/) was used to convert the coordinates between different reference genome versions, and Gffread [59] was used to interconvert the GFF3 and GTF file formats. We also integrated a range of additional datasets that intersect functionally with Ψ modifications, including RNA-binding protein (RBP)-binding datasets from POSTAR3 [60], genome-wide association study (GWAS) datasets from the GWAS Atlas database [61], small open reading frame (sORF) datasets from the PsORF database (http://psorf.whu.edu.cn), expression quantitative trait locus (eQTL) datasets from the AtMAD [62] and Rice-eQTL (http://riceqtl.ncpgr.cn/) databases, R-loop datasets from the R-loopAtlas database [63], alternative polyadenylation (APA) site datasets from the PlantAPAdb database [64], conservation datasets from the PlantRegMap database [65], and RNA structural datasets, namely RNA G-quadruplex (rG4) data from the G4Atlas database [66] and transcriptome-scale RNA secondary-structure probing data from the RNA Atlas of Structure Probing (RASP) database [67] (Table S3).

In addition to the data curated from the literature and existing databases, we generated predictions from DRS. FAST5 datasets were retrieved from public repositories for *A. thaliana* [European Nucleotide Archive (ENA): PRJEB53881], *Glycine max* (ENA: PRJEB44525), *Oryza sativa* (NCBI: PRJNA943203), *Populus trichocarpa* (NCBI: PRJNA672182), and *Zea mays* (NCBI: PRJNA635654). The raw FAST5 signals were first converted to FASTQ format using Guppy (v6.0.1; https://community.nanoporetech.com/), after which Ψ modification sites were predicted with NanoPsu. A probability threshold of 0.9 was applied to retain high-confidence sites, yielding a reliable set of Ψ sites across these plant species. The NanoPsu prediction files were then annotated using customized scripts adapted from our in-house RNAmod pipeline [68], and the annotation results are displayed on the “Browse by DRS” page of Ψ-Atlas.

### Enhanced web interface and usage in Ψ-Atlas

The database interface of Ψ-Atlas has been meticulously designed to provide a comprehensive, rapid, and user-friendly centralized knowledge repository for Ψ site research, allowing users to swiftly query, customize searches, and freely download all gathered datasets. Four primary modules are available in Ψ-Atlas: “Browse”, “Search”, “JBrowse”, and “Tools”.

**Figure 3:**
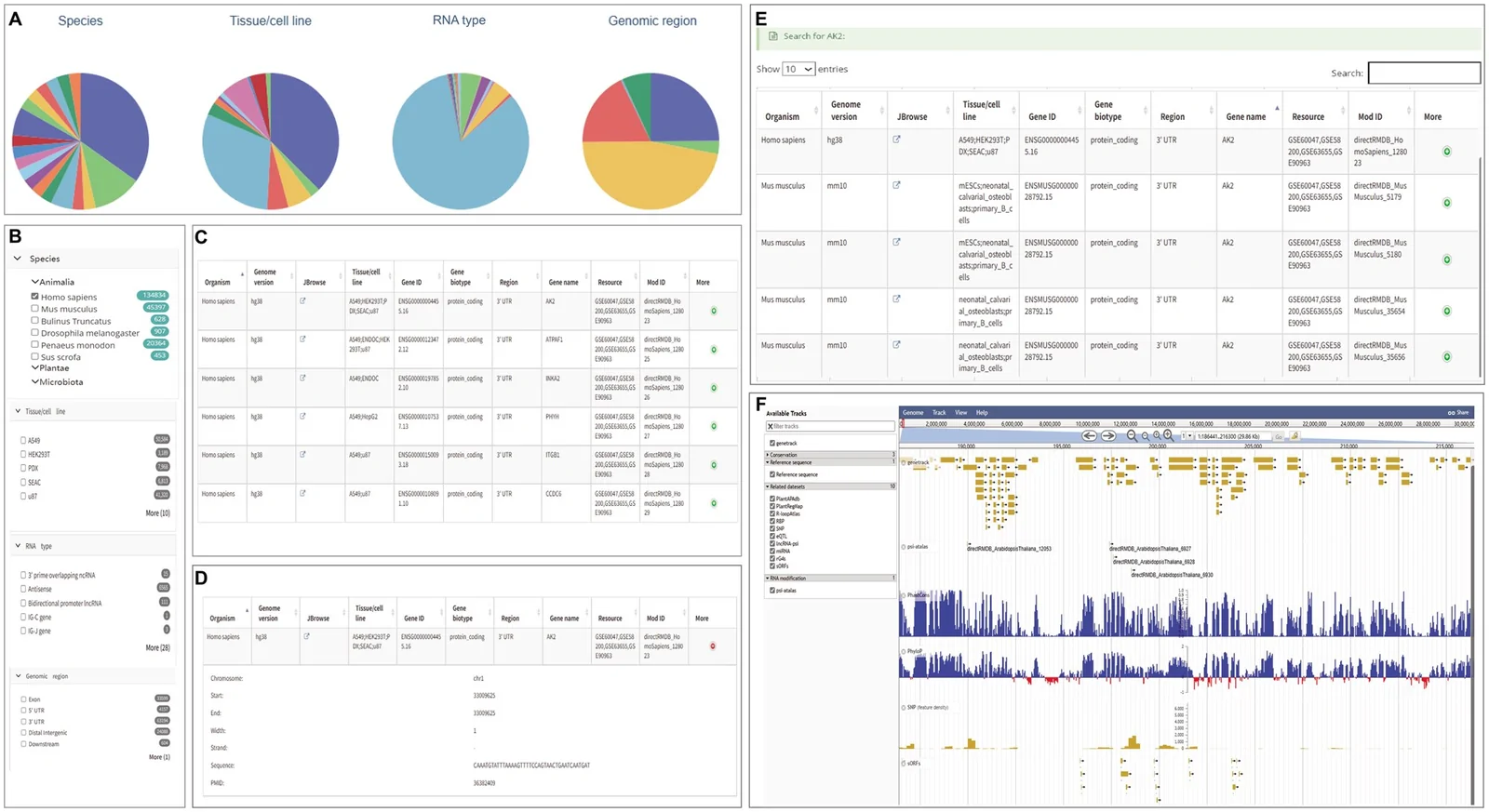
The Browse, Search, and JBrowse modules of Ψ-Atlas The collected datasets are divided into four groups based on species, tissue/cell line, RNA type, and genomic region. **A**. Users can view the data distribution through pie charts. **B**. Users can select categories of interest to update the table and query the data. **C**. The table displays basic information of the Ψ sites. **D**. Clicking “More” to obtain detailed information. **E**. Results of the “Search” module. **F**. Visualization of JBrowse data displaying tracks of all Ψ modification sites and other publicly available genome annotations.

The “Browse” module is divided into three main dataset groups: (1) Browse of NGS datasets, (2) Browse of DRS datasets, and (3) Browse of other datasets, allowing users to easily distinguish between different sources of Ψ modification data. Both the “Browse of NGS datasets” and “Browse of DRS datasets” groups can be filtered by species, tissue/cell line, RNA type, and genomic region. Users can query Ψ modification loci through two distinct interfaces: “Browse by species” or “Browse by tissue/cell line” (**Figure 3**A and B). In addition to basic information for each Ψ site (*e.g.*, genome version, gene ID, and gene name), users can obtain detailed genomic location and sequence information through the “More” option (Figure 3C and D). The “Browse of NGS datasets” group includes a total of 41 species, comprising 12 animals, 9 plants, and 20 microorganisms, while the “Browse of DRS datasets” group includes 18 species, comprising 6 animals, 5 plants, and 7 microorganisms. The “Browse of other datasets” group includes two data tables: (1) Ψ sites validated by other methods and (2) other Ψ site-related datasets. The other datasets contain Ψ sites identified through experimental methods such as liquid chromatography–tandem mass spectrometry (LC–MS/MS) and 2D-TLC.

The “Search” module allows users to quickly retrieve comprehensive information on Ψ modifications by submitting gene IDs, gene names, or PubMed IDs. Moreover, quickly search function is available on the Ψ-Atlas homepage for single-query searches (Figure 3E).

The “JBrowse” module is integrated into a swift and scalable genome browser constructed with JavaScript and HTML5, enabling visualization of all Ψ modification sites and other publicly available genome annotations, such as RBP-binding sites, microRNA (miRNA) targets, splice sites, single-nucleotide variations (SNVs), and single-nucleotide polymorphisms (SNPs) (Figure 3F).

The “Tools” module consists of three sections: (1) “PsiVar” is used to identify the potential variant effects of Ψ modifications; (2) “PsiFinder” is a high-precision Ψ predictor based on deep learning, capable of identifying Ψ across various species; (3) “Retrieve results” allows users to quickly check the status of their submitted analysis tasks by job ID.

The other modules include: (1) the “Download” module, which provides datasets for various sequencing methods and species, enabling users to download data by “Species” and “Data type”, as well as information related to Ψ modifications in human diseases; (2) the “Links” module, which offers links to numerous software tools and databases for Ψ modifications; and (3) the “Help” module, which provides a series of tutorials and annotations through demonstrations.

### Web-based analytical tools developed in Ψ-Atlas

**Figure 4:**
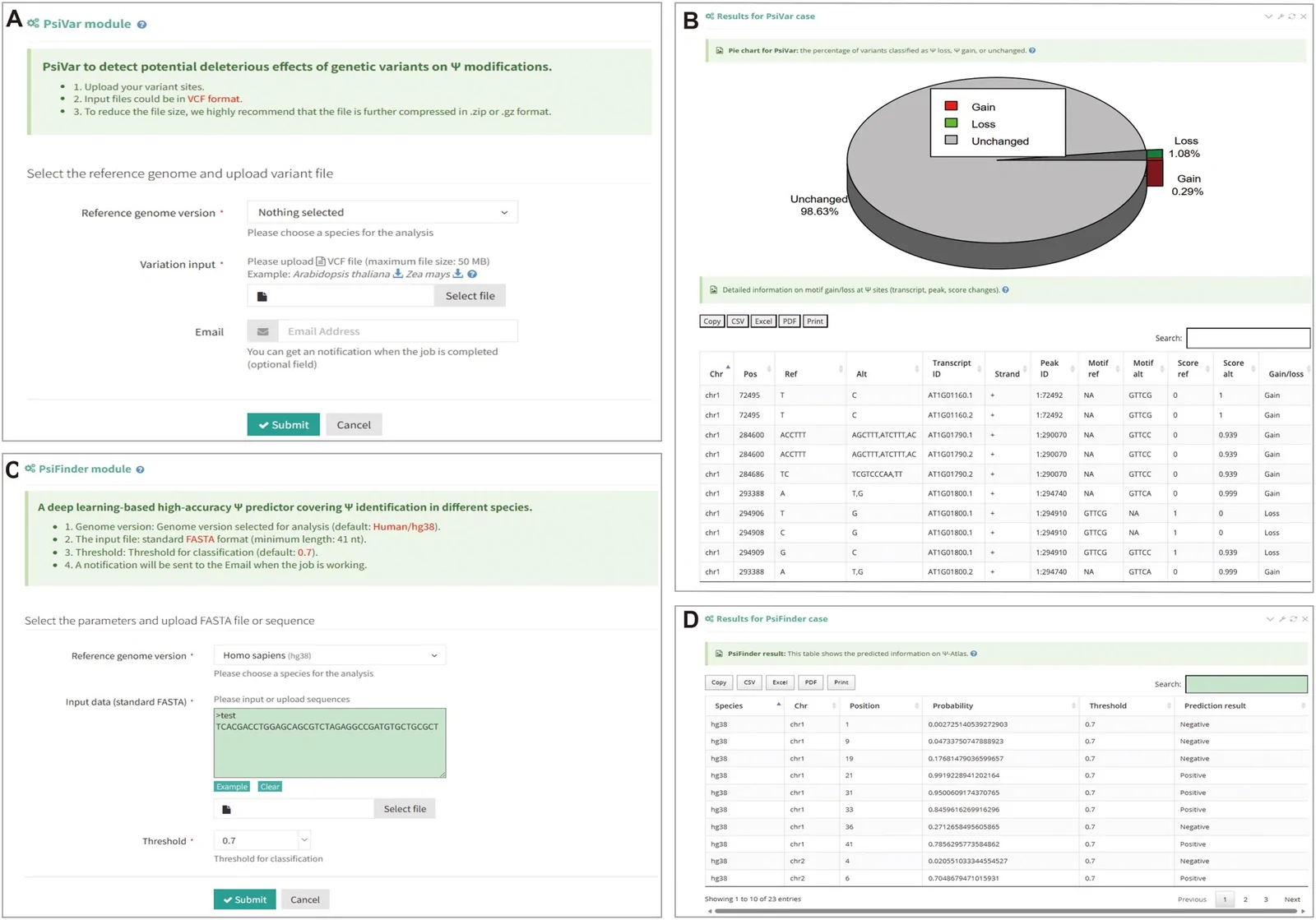
Web-based analytical tools **A**. PsiVar is used to identify the potential variant effects of Ψ modifications. **B**. Results of PsiVar showing the distribution of variants classified as Ψ loss, Ψ gain, or unchanged. **C**. PsiFinder is a high-precision Ψ predictor based on deep learning, capable of identifying Ψ across various species. **D**. Predicted results of PsiFinder.

PsiVar was designed to detect the potential effects of genetic variants on Ψ modifications. Variant files in standard VCF format serve as the input, and the uploaded SNPs are analyzed to assess their impact on the curated Ψ sites of the corresponding species. Upon submission, the queuing system assigns a job ID, which users can use to monitor analysis progress on the current-analysis page or to retrieve results from the “Tools” module; users may also opt to receive an email notification once the job is complete (**Figure 4**A). After evaluating the sequence characteristics of each site, PsiVar classifies the variants as Ψ loss, Ψ gain, or unchanged. The results are returned as a pie chart showing the distribution of these three categories, together with a detailed table reporting variant position, transcript ID, peak ID, reference motif, mutated motif, and the scores used to determine Ψ loss or gain. As an example, PsiVar analyzed the sequence characteristics of the first 10,000 mutated sites from the *A. thaliana* SNP data of the EnsemblPlants database (https://ftp.ensemblgenomes.ebi.ac.uk/pub/plants/release-60/variation/vcf/arabidopsis_thaliana/); approximately 1.08% of the mutated sites were classified as Ψ loss variants and 0.29% as Ψ gain variants (Figure 4B; Table S4).

PsiFinder is a deep-learning tool developed to accurately predict putative Ψ sites from user-uploaded sequences in standard FASTA format. It employs a convolutional neural network (CNN) trained and tested on datasets derived from human and mouse transcriptomes. For each candidate site (T-base site), a 41-nt window consisting of the site itself plus 20 nt upstream and 20 nt downstream is used as input. Transcripts containing Ψ-Seq-validated Ψ modification sites were designated as positive samples, whereas randomly selected T-base sites together with their flanking 20-nt sequences served as negative samples. Equal numbers of positive and negative samples were then drawn from the full pool of 41-nt sequences to construct a balanced dataset. This dataset was randomly partitioned into training, validation, and test sets at ratios of 80%, 10%, and 10%, respectively, with the training set comprising 14,520 samples. A deep neural network was trained on each dataset, and the resulting binary classifiers were combined into an ensemble model, with the final prediction score obtained by averaging the scores across all classifiers. In operation, the uploaded sequences are one-hot encoded and passed to the pre-trained model: the input is processed through two convolutional layers, followed by a normalization layer and a Rectified Linear Unit (ReLU) activation, and then mapped to the output through a pooling layer and a fully connected layer (Figure 4C). As an example, a user may upload a randomly selected 41-nt human FASTA sequence, specify the corresponding species, and set a prediction threshold (default 0.7). The prediction information is displayed in a table that includes species, chromosome, position, probability, threshold, and prediction result, allowing users to assess the likelihood of potential Ψ modification sites (Figure 4D).

## Discussion

Currently, several databases and web servers integrate existing Ψ modification sites; however, these databases are not specifically designed for Ψ modifications and cover a limited number of species and Ψ sites (**Table 1**). For example, RMDisease (v2.0) [39] identified a total of 1,366,252 RNA modification-associated variants that may affect 16 different types of RNA modification in 20 organisms, while only 4 species (human, mouse, yeast, and *Arabidopsis*) contain a small number of identified Ψ modification sites. DirectRMDB [38] is an Oxford Nanopore Technologies (ONT)-based database that includes 16 types of RNA modification and a total of 904,712 modification sites in 25 species, but it only contains DRS datasets and does not include Ψ sites identified by experimental or NGS-based approaches. EPITOMY (https://epitomy.luddy.indianapolis.iu.edu) only focuses on human and mouse and has limited Ψ research. PRMD [41] is a convenient and comprehensive database of plant RNA modifications, but its analysis modules are exclusively focused on plant species and predominantly cover m^6^A modifications, with very limited data on Ψ modifications. RMBase (v3.0) [40] is a comprehensive and convenient platform for the efficient study of RNA modifications from large amounts of epitranscriptomic high-throughput sequencing data. It provides multiple interfaces and web-based tools to integrate 73 types of RNA modification across 62 species; however, Ψ modification studies are not the primary focus of this database. In summary, none of these databases was developed specifically for Ψ modification research, and based on the urgent need for a database focused on the integration of Ψ modification datasets, we developed Ψ-Atlas by collecting and processing all previously published Ψ modification data from 77 species. Additionally, Ψ-Atlas incorporates convenient analysis modules such as PsiVar and PsiFinder for data analyses, as well as embedding JBrowse for data visualization. Moreover, Ψ-Atlas intuitively displays the related datasets from previous studies and other resources such as SNVs, GWAS, RNA secondary structures, rG4 structures, and RBP-binding sites.

**Table 1:** Comparison of Ψ-Atlas with existing Ψ modification databases

	Ψ-Atlas	RMDisease (v2.0)	EPITOMY	DirectRMDB	RMBase (v3.0)	PRMD
**Data sources**						
No. of species	77	4	2	25	62	20
Experimentally validated sites	Yes	No	Yes	No	Yes	No
NGS	Yes	Yes	Yes	No	Yes	Yes
DRS (nanopore)	Yes	No	No	Yes	No	Yes
**Other functional annotations**						
RBP-binding site	Yes	Yes	No	Yes	Yes	Yes
RNA structure	Yes	Yes	No	No	Yes	Yes
miRNA target site	Yes	Yes	No	Yes	Yes	Yes
SNP	Yes	Yes	No	Yes	Yes	Yes
eQTL	Yes	No	No	No	No	Yes
GWAS site	Yes	Yes	No	No	No	Yes
sORF	Yes	No	No	No	No	Yes
APA site	Yes	No	No	No	No	Yes
**Tools and visualization**						
Ψ predictor	Yes	Yes	No	No	No	No
Variation effects on modifications	Yes	No	No	No	No	Yes
JBrowse	Yes	Yes	No	Yes	Yes	Yes
**The ways to query datasets**						
Gene ID	Yes	Yes	Yes	No	No	Yes
Gene name	Yes	Yes	Yes	Yes	No	Yes
Job ID	Yes	No	No	No	No	Yes
PubMed ID	Yes	No	Yes	No	No	Yes
Link	https://rnainformatics.org.cn/PsiAtlas	http://www.rnamd.org/rmdisease2	https://epitomy.luddy.indianapolis.iu.edu	http://www.rnamd.org/directRMDB	https://rna.sysu.edu.cn/rmbase3/	http://bioinformatics.sc.cn/PRMD

*Note*: Ψ, pseudouridine; NGS, next-generation sequencing; DRS, direct RNA sequencing; RBP, RNA-binding protein; miRNA, microRNA; SNP, single-nucleotide polymorphism; eQTL, expression quantitative trait locus; GWAS, genome-wide association study; sORF, small open reading frame; APA, alternative polyadenylation.

## Conclusion

Among over 170 identified ribonucleotide modifications, Ψ modification is one of the most abundant [1]. Several studies have demonstrated that Ψ modifications in RNA influence growth, development, and physiological and pathological processes across diverse species [4,8,20]. Although several published databases have integrated existing sequencing datasets and Ψ-related information, a comprehensive and user-friendly database specifically dedicated to Ψ modification is still lacking. Therefore, we developed Ψ-Atlas, spanning 77 species, to facilitate research on Ψ modifications. Currently, Ψ-Atlas integrates 554,895 Ψ modification sites from NGS, DRS, and other experimental chemical technologies. With continuous advancements in high-throughput sequencing technologies, the availability of Ψ-related sequencing data is rapidly increasing. To address this, we will regularly update the Ψ-Atlas database with newly published high-throughput sequencing data to provide more comprehensive information on Ψ modifications. Besides, we plan to develop more tools for functional analysis of Ψ-Atlas datasets, facilitating research and progress in this field.

## Code availability

The code, PsiVar, and PsiFinder generated in this study have been submitted to BioCode at the National Genomics Data Center (NGDC), China National Center for Bioinformation (CNCB) (BioCode: BT007923, BT007924, and BT007926), which are publicly accessible at https://ngdc.cncb.ac.cn/biocode/tool/BT007923, https://ngdc.cncb.ac.cn/biocode/tool/BT007924, and https://ngdc.cncb.ac.cn/biocode/tool/BT007926, respectively.

## Supplementary Material

qzag004_Supplementary_Data

## Data Availability

Ψ-Atlas is freely available at https://rnainformatics.org.cn/PsiAtlas. It has also been submitted to Database Commons [69] at the NGDC, CNCB, which is publicly accessible at https://ngdc.cncb.ac.cn/databasecommons/database/id/10284.

## CRediT author statement

**Xiaochen Wang:** Investigation, Methodology, Data curation, Writing – original draft, Writing – review & editing. **Jinjing Luo:** Methodology, Data curation, Formal analysis, Software, Writing – original draft. **Xiaoqiang Lang:** Investigation, Methodology, Data curation, Formal analysis, Writing – original draft. **Yongqing Ling:** Software, Formal analysis. **Yiming Zhou:** Data curation, Formal analysis. **Guoxian Liu:** Investigation, Data curation. **Xiangye Chen:** Investigation, Data curation. **Yibo Chen:** Writing – review & editing, Supervision. **Yingshun Zhou:** Writing – review & editing, Supervision. **Yi Cao:** Writing – review & editing. **Zhonghui Zhang:** Writing – review & editing. **Changjun Ding:** Supervision, Funding acquisition. **Demeng Chen:** Methodology, Supervision, Funding acquisition. **Qi Liu:** Conceptualization, Methodology, Project administration, Resources, Supervision, Funding acquisition, Writing – review & editing. All authors have read and approved the final manuscript.
