# Supplementary material for: Ψ-Atlas: An Integrated Atlas for Pseudouridine Epitranscriptome
Source: Genomics Proteomics Bioinformatics. 2026 Jan 23;24(2):qzag004. doi: 10.1093/gpbjnl/qzag004 (PMC13529368; doi:10.1093/gpbjnl/qzag004)
Supplement: Supplementary file 1 [file qzag004_supplementary_data.zip › Table S1.docx]

**Table S1 Collection of Ψ modification sites in Ψ-Atlas**

| **Species** | **NGS** | **DRS** | **Other research sites** | **Total** | **Genome version** |
| --- | --- | --- | --- | --- | --- |
| *Homo sapiens* | 126055 | 136565 | 38715 | 301,335 | hg38 |
| *Mus musculus* | 32358 | 45397 | 36206 | 113,961 | mm10 |
| *Saccharomyces cerevisiae* | 2484 | 59 | 9727 | 12,270 | sacCer3(R64) |
| *Arabidopsis thaliana* | 76 | 10431 | 2 | 10,509 | TAIR10 |
| *Brassica napus* | 175 | / | / | 175 | PRJEB5043v1 |
| *Bacillus subtilis* | 99 | 3 | 17 | 119 | ASM69118v1 |
| *Bombyx mori* | 185 | / | / | 185 | ASM15162v1 |
| *Bos taurus* | 381 | / | / | 381 | bosTau9 |
| *Bipolaris sorokiniana* | / | / | 3772 | 3772 | Cocsa1 |
| *Bulinus truncatus* | / | 628 | / | 628 | Btru.v1 |
| *Caenorhabditis elegans* | 296 | / | 74 | 370 | ce11 |
| *Candida albicans* | 3 | / | / | 3 | Cand_albi12Cv2 |
| *Chlamydomonas reinhardtii* | 30 | / | / | 30 | CHLRE3 |
| *Cyanidioschyzon merolae* | / | / | 6 | 6 | / |
| *Cucumis melo* | / | 10683 | / | 10,683 | ASM31304v1 |
| *Cucumis sativus* | 34 | / | / | 34 | ASM407v2 |
| *Chikungunya virus* | / | / | 180 | 180 | ViralProj14998 |
| *Danio rerio* | 5 | / | / | 5 | danRer11(GRCz11) |
| *Deinococcus radiodurans* | / | / | 4 | 4 | / |
| *Drosophila melanogaster* | 614 | 907 | 2640 | 4161 | BDGP6 |
| *Enterobacteria phage T4* | 14 | / | / | 14 | ViralProj14044 |
| *Enterobacteria phage T5* | 9 | / | / | 9 | ASM292140v1 |
| *Escherichia coli* | 206 | 392 | 189 | 787 | ASM584v2 |
| *Gallus gallus* | 36 | / | / | 36 | GRCg6a |
| *Geobacillus stearothermophilus* | 11 | / | / | 11 | ASM74998v1 |
| *Glycine max* | 4 | 7125 | / | 7129 | glyMax2 |
| *Helianthus annuus* | / | 2083 | / | 2083 | HanXRQr2.0-SUNRISE |
| *Halococcus morrhuae* | 1 | / | / | 1 | ASM33669v1 |
| *Halobacterium salinarum* | 17 | / | / | 17 | ASM680v1 |
| *Haloarcula marismortui* | / | / | 3 | 3 | / |
| *Halobacterium halobium* | / | / | 4 | 4 | / |
| *Haloferax volcanii* | 79 | 202 | 13 | 294 | ASM2568v1 |
| *Leishmania major* | / | / | 37 | 37 | / |
| *Mesocricetus auratus* | 5 | / | / | 5 | MesAur1.0 |
| *Mycoplasma capricolum* | 38 | / | / | 38 | ASM83033v1 |
| *Marchantia polymorpha* | / | / | 1 | 1 | / |
| *Methanocaldococcus jannaschii* | / | / | 2 | 2 | / |
| *Mycobacterium smegmatis* | 1 | / |  | 1 | ASM76770v1 |
| *Mycoplasma* *mycoides* | 4 | / | / | 4 | ASM25307v1 |
| *Nakaseomyces nivariensis* | / | 599 | / | 599 | JHU_Cniv_v1 |
| *Neurospora crassa* | 36 | / | / | 36 | NC12 |
| *Nicotiana tabacum* | 134 | / | / | 134 | Ntab_TN90 |
| *Oryctolagus cuniculus* | 224 | / | / | 224 | OryCun2.0 |
| *Ovis aries* | 35 | / | / | 35 | Oarv3 |
| *Oryza sativa* | / | 1343 |  | 1343 | IRGSP-1 |
| *Penaeus monodon* | / | 20364 | / | 20,364 | NSTDA_Pmon_1 |
| *Pichia jadinii* | 108 | / | / | 108 | Cybja1 |
| *Pyrococcus furiosus* | / | 207 | 1 | 208 | ASM824508v1 |
| *Photinus pyralis* | / | 2 | / | 2 | Ppyr1.3 |
| *Phaseolus vulgaris* | 78 | / | / | 78 | PhaVulg1 |
| *Physcomitrium patens* | / | 11308 | / | 11,308 | Phypa |
| *Populus trichocarpa* | / | 10552 | / | 10,552 | Pop_tri_v3 |
| *Rattus norvegicus* | 1317 | / | / | 1317 | rn6(Rnor_6.0) |
| *Rhodospirillum rubrum* | / | 2 | / | 2 | ASM1308v1 |
| *Salmonella typhimurium* | 19 | / | / | 19 | ASM553737v1 |
| *Asteroidea* | / | / | 1 | 1 | / |
| *Streptomyces coelicolor A3(2)* | 1 | / |  | 1 | ASM20383v1 |
| *Schizosaccharomyces pombe* | 41 | / | / | 41 | ASM294v2 |
| *Solanum tuberosum* | 46 | / | / | 46 | SolTub3 |
| *Streptomyces griseus* | 4 | / | / | 4 | ASM93222v1 |
| *Spinacia oleracea* | 89 | / | / | 89 | ASM200726v1 |
| *Spiroplasma citri* | 3 | / | / | 3 | ASM188685v1 |
| *Synechococcus* sp. | 2 | / | / | 2 | ASM1948v1 |
| *Synechocystis* sp. | 1 | / | / | 1 | ASM34078v1 |
| *Sulfolobus acidocaldarius* | / | / | 6 | 6 | / |
| *Sus scrofa* | / | 453 | / | 453 | Sscrofa11.1 |
| *Synechococcus elongatus* | 8 | / | / | 8 | ASM1006v1 |
| *Thermoplasma acidophilum* | / | / | 2 | 2 | / |
| *Thermoplasma acidophilum* | 10 | / | / | 10 | ASM19591v1 |
| *Thermococcus kodakarensis* | / | / | 3 | 3 | / |
| *Trypanosoma brucei* | / | / | 380 | 380 | / |
| *Tetrahymena thermophila* | 165 | / | / | 165 | JCVI_TTA1_2 |
| *Thermus thermophilus* | 19 | / | 55 | 74 | ASM812v1 |
| *Tymovirus* | / | / | 2 | 2 | / |
| *Toxoplasma gondii* | / | 11397 | / | 11,397 | TGA4 |
| *Xenopus laevis* | 104 | / | 15 | 119 | Xenopus_laevisv2 |
| *Vibrio cholerae* | / | / | 1 | 1 | / |
| *Zea mays* | 15 | 26456 | / | 26,471 | Zm-B73-REFERENCE-NAM-5.0 |

*Note*: “/” represents no data available. NGS, next-generation sequencing; DRS, direct RNA sequencing.
