# Supplementary material for: Ψ-Atlas: An Integrated Atlas for Pseudouridine Epitranscriptome
Source: Genomics Proteomics Bioinformatics. 2026 Jan 23;24(2):qzag004. doi: 10.1093/gpbjnl/qzag004 (PMC13529368; doi:10.1093/gpbjnl/qzag004)
Supplement: Supplementary file 1 [file qzag004_supplementary_data.zip › Table S2.docx]

**Table S2 Collection of Ψ modifications datasets resource**

| **Number** | **Title** | **PMID** | **DOI** | **Raw data** |
| --- | --- | --- | --- | --- |
| 1 | Quantitative profiling of pseudouridylation landscape in the human transcriptome | 36997645 | 10.1038/s41589-023-01304-7 | GSE212210 & GSE60047 & GSE58200 & GSE63655 & GSE179798 |
| 2 | Pseudouridines on Trypanosoma brucei mRNAs are developmentally regulated: Implications to mRNA stability and protein binding | 34165831 | 10.1111/mmi.14774 | PRJNA656693 |
| 3 | Pseudouridine profiling reveals regulated mRNA pseudouridylation in yeast and human cells | 25192136 | 10.1038/nature13802 | GSE58200 |
| 4 | Chemical pulldown reveals dynamic pseudouridylation of the mammalian transcriptome | 26075521 | 10.1038/nchembio.1836 | GSE63655 |
| 5 | Transcriptome-wide mapping of pseudouridines: pseudouridine synthases modify specific mRNAs in S. cerevisiae | 25353621 | 10.1371/journal.pone.0110799 | GSE60445 |
| 6 | Pseudouridines on Trypanosoma brucei spliceosomal small nuclear RNAs and their implication for RNA and protein interactions | 31147702 | 10.1093/nar/gkz477 | PRJNA476671 |
| 7 | Transcriptome-wide mapping reveals widespread dynamic-regulated pseudouridylation of ncRNA and mRNA | 25219674 | 10.1016/j.cell.2014.08.028 | GSE60047 |
| 8 | Complete chemical structures of human mitochondrial tRNAs | 32859890 | 10.1038/s41467-020-18068-6 | PRJNA638467 & GSE150439 |
| 9 | Landscape of the complete RNA chemical modifications in the human 80S ribosome | 30202881 | 10.1093/nar/gky811 | / |
| 10 | Quantitative profiling of pseudouridylation dynamics in native RNAs with nanopore sequencing | 33986546 | 10.1038/s41587-021-00915-6 | PRJNA511582 & PRJNA549001 & PRJNA695584 & GSE148603 & PRJEB37798 & PRJEB41495 |
| 11 | Interferon inducible pseudouridine modification in human mRNA by quantitative nanopore profiling | 34872593 | 10.1186/s13059-021-02557-y | / |
| 12 | Mapping of pseudouridine residues on cellular and viral transcripts using a novel antibody-based technique | 34376564 | 10.1261/rna.078940.121 | GSE172136 |
| 13 | Direct Determination of Pseudouridine in RNA by Mass Spectrometry Coupled with Stable Isotope Labeling | 32662983 | 10.1021/acs.analchem.0c02122 | / |
| 14 | A mass spectrometry-based method for direct determination of pseudouridine in RNA | 26673725 | 10.1093/nar/gkv1462 | / |
| 15 | H/ACA small RNA dysfunctions in disease reveal key roles for /coding RNA modifications in hematopoietic stem cell differentiation | 23707062 | 10.1016/j.celrep.2013.04.030 | / |
| 16 | TRUB1 is the predominant pseudouridine synthase acting on mammalian mRNA via a predictable and conserved code | 28073919 | 10.1101/gr.207613.116 | GSE90851 & GSE60047 & GSE58200 & GSE63655 |
| 17 | Transcriptome-wide profiling of multiple RNA modifications simultaneously at single-base resolution | 30872485 | 10.1073/pnas.1817334116 | GSE90963 & https://github.com/HuntsmanCancerInstitute/RBSSeqTools. |
| 18 | A dual-purpose polymerase engineered for direct sequencing of pseudouridine and queuosine | 36971106 | 10.1093/nar/gkad177 | GSE217198 & GSE216921 |
| 19 | HydraPsiSeq: a method for systematic and quantitative mapping of pseudouridines in RNA | 32976574 | 10.1093/nar/gkaa769 | / |
| 20 | DirectRMDB: a database of post-transcriptional RNA modifications unveiled from direct RNA sequencing technology | 36382409 | 10.1093/nar/gkac1061 | GSE60047 & GSE58200 & GSE63655 & GSE90963 |
| 21 | Semi-quantitative detection of pseudouridine modifications and type I/II hypermodifications in human mRNAs using direct long-read sequencing | 36658122 | 10.1038/s41467-023-35858-w. | PRJNA777450 |
| 22 | Transcriptome-wide analysis of pseudouridylation in Drosophila melanogaster | 36534986 | 10.1093/g3journal/jkac333 | GSE213312 |
| 23 | BID-seq: The Quantitative and Base-Resolution Sequencing Method for RNA Pseudouridine | 36525588 | 10.1021/acschembio.2c00881 | GSE179798 |
| 24 | Intron-Dependent or Independent Pseudouridylation of Precursor tRNA Containing Atypical Introns in Cyanidioschyzon merolae | 36292915 | 10.3390/ijms232012058 | / |
| 25 | Sequential action of a tRNA base editor in conversion of cytidine to pseudouridine | 36220828 | 10.1038/s41467-022-33714-x | / |
| 26 | Human TRUB1 is a highly conserved pseudouridine synthase responsible for the formation of Ψ55 in mitochondrial tRNAAsn, tRNAGln, tRNAGlu and tRNAPro | 36018806 | 10.1093/nar/gkac698 | / |
| 27 | Identification and functional implications of pseudouridine RNA modification on small /coding RNAs in the mammalian pathogen Trypanosoma brucei | 35714765 | 10.1016/j.jbc.2022.102141 | PRJNA797695 & PRJNA476671 & PRJNA630014 |
| 28 | RNA modification mapping with JACUSA2 | 35578346 | 10.1186/s13059-022-02676-0 | PRJNA824272 & SRP132322 |
| 29 | Pseudouridines have context-dependent mutation and stop rates in high-throughput sequencing | 29683381 | 10.1080/15476286.2018.1462654 | SRP132322 |
| 30 | The Arabidopsis Mitochondrial Pseudouridine Synthase Homolog FCS1 Plays Critical Roles in Plant Development | 35560171 | 10.1093/pcp/pcac060 | / |
| 31 | Penguin: A tool for predicting pseudouridine sites in direct RNA nanopore sequencing data | 35182749 | 10.1016/j.ymeth.2022.02.005 | / |
| 32 | DARNED: a DAtabase of RNa EDiting in humans | 20547637 | 10.1093/bioinformatics/btq285 | / |
| 33 | RMBase v2.0: deciphering the map of RNA modifications from epitranscriptome sequencing data | 29040692 | 10.1093/nar/gkx934 | / |
| 34 | Pseudouridine: the fifth RNA nucleotide with renewed interests | 27348156 | 10.1016/j.cbpa.2016.06.014 | / |
| 35 | BID-seq for transcriptome-wide quantitative sequencing of mRNA pseudouridine at base resolution | 37968414 | 10.1038/s41596-023-00917-5 | GSE238245 |
| 36 | Analysis of pseudouridines and other RNA modifications using HydraPsiSeq protocol | 34481083 | 10.1016/j.ymeth.2021.08.008 | / |
| 37 | SnoRNA guide activities: real and ambiguous | 34385348 | 10.1261/rna.078916.121 | / |
| 38 | Pseudouridine RNA modification detection and quantification by RT-PCR | 34020036 | 10.1016/j.ymeth.2021.05.010 | / |
| 39 | Mammalian nuclear TRUB1, mitochondrial TRUB2, and cytoplasmic PUS10 produce conserved pseudouridine 55 in different sets of tRNA | 33023933 | 10.1261/rna.076810.120 | / |
| 40 | tRNA elbow modifications affect the tRNA pseudouridine synthase TruB and the methyltransferase TrmA | 32385137 | 10.1261/rna.075473.120 | / |
| 41 | Loss of m1acp3Ψ Ribosomal RNA Modification Is a Major Feature of Cancer | 32375039 | 10.1016/j.celrep.2020.107611 | / |
| 42 | Sensitive and quantitative probing of pseudouridine modification in mRNA and long /coding RNA | 31227565 | 10.1261/rna.072124.119 | / |
| 43 | Impact of intron removal from tRNA genes on Saccharomyces cerevisiae | 30997502 | 10.1093/nar/gkz270 | / |
| 44 | The human ortholog of archaeal Pus10 produces pseudouridine 54 in select tRNAs where its recognition sequence contains a modified residue | 30530625 | 10.1261/rna.068114.118 | / |
| 45 | Post-transcriptional pseudouridylation in mRNA as well as in some major types of /coding RNAs | 30414851 | 10.1261/rna.068114.118 | / |
| 46 | Pseudouridylation meets next-generation sequencing | 26968262 | 10.1016/j.ymeth.2016.03.001 | / |
| 47 | Contribution of protein Gar1 to the RNA-guided and RNA-independent rRNA:Ψ-synthase activities of the archaeal Cbf5 protein | 30218085 | 10.1038/s41598-018-32164-0 | / |
| 48 | Directed Evolution of Heterologous tRNAs Leads to Reduced Dependence on Post-transcriptional Modifications | 29694026 | 10.1021/acssynbio.7b00421 | / |
| 49 | Dual nature of pseudouridylation in U2 snRNA: Pus1p-dependent and Pus1p-independent activities in yeasts and higher eukaryotes | 28432181 | 10.1261/rna.061226.117 | / |
| 50 | Small methyltransferase RlmH assembles a composite active site to methylate a ribosomal pseudouridine | 28428565 | 10.1038/s41598-017-01186-5 | / |
| 51 | Role of Pseudouridine Formation by Deg1 for Functionality of Two Glutamine Isoacceptor tRNAs | 28134782 | 10.3390/biom7010008 | / |
| 52 | Pseudouridine in the Anticodon of Escherichia coli tRNATyr(QΨA) Is Catalyzed by the Dual Specificity Enzyme RluF | 27551044 | 10.1074/jbc.M116.747865 | / |
| 53 | A Deafness- and Diabetes-associated tRNA Mutation Causes Deficient Pseudouridinylation at Position 55 in tRNAGlu and Mitochondrial Dysfunction | 27519417 | 10.1074/jbc.M116.739482 | / |
| 54 | Pseudouridines in U2 snRNA stimulate the ATPase activity of Prp5 during spliceosome assembly | 26873591 | 10.15252/embj.201593113 | / |
| 55 | The presence of the ACA box in archaeal H/ACA guide RNAs promotes atypical pseudouridylation | 31919243 | 10.1261/rna.073734.119 | / |
| 56 | Structure modulation of helix 69 from Escherichia coli 23S ribosomal RNA by pseudouridylations | 24371282 | 10.1093/nar/gkt1329 | / |
| 57 | Role of forefinger and thumb loops in production of Ψ54 and Ψ55 in tRNAs by archaeal Pus10 | 23898217 | 10.1261/rna.039230.113 | / |
| 58 | Discovery of Pyrobaculum small RNA families with atypical pseudouridine guide RNA features | 22282340 | 10.1261/rna.031385.111 | / |
| 59 | The archaeal COG1901/DUF358 SPOUT-methyltransferase members, together with pseudouridine synthase Pus10, catalyze the formation of 1-methylpseudouridine at position 54 of tRNA | 22274953 | 10.1261/rna.030841.111 | / |
| 60 | Human tRNA(Lys3)(UUU) is pre-structured by natural modifications for cognate and wobble codon binding through keto-enol tautomerism. | 22227389 | 10.1016/j.jmb.2011.12.048 | / |
| 61 | Pseudouridine synthase 1: a site-specific synthase without strict sequence recognition requirements. | 22102571 | 10.1093/nar/gkr1017 | / |
| 62 | Comparison of solution conformations and stabilities of modified helix 69 rRNA analogs from bacteria and human | 21858779 | 10.1002/bip.21706 | / |
| 63 | Pseudouridine formation in archaeal RNAs: The case of Haloferax volcanii | 21628430 | 10.1261/rna.2712811 | / |
| 64 | Pseudouridylation of 23S rRNA helix 69 promotes peptide release by release factor RF2 but not by release factor RF1 | 21281690 | 10.1016/j.biochi.2010.12.018 | / |
| 65 | U2 snRNA is inducibly pseudouridylated at novel sites by Pus7p and snR81 RNP | 21131909 | 10.1038/emboj.2010.316 | / |
| 66 | Pseudouridine at position 55 in tRNA controls the contents of other modified nucleotides for low-temperature adaptation in the extreme-thermophilic eubacterium Thermus thermophilus | 21097467 | 10.1093/nar/gkq1180 | / |
| 67 | Inactivation of the RluD pseudouridine synthase has minimal effects on growth and ribosome function in wild-type Escherichia coli and Salmonella enterica | 21037010 | 10.1128/JB.00970-10 | / |
| 68 | Subribosomal particle analysis reveals the stages of bacterial ribosome assembly at which rRNA nucleotides are modified | 20719918 | 10.1261/rna.2160010 | / |
| 69 | The structure of the human tRNALys3 anticodon bound to the HIV genome is stabilized by modified nucleosides and adjacent mismatch base pairs | 19324888 | 10.1093/nar/gkp187 | / |
| 70 | Identification of pseudouridine methyltransferase in Escherichia coli | 18755836 | 10.1261/rna.1186608 | / |
| 71 | YbeA is the m3Psi methyltransferase RlmH that targets nucleotide 1915 in 23S rRNA | 18755835 | 10.1261/rna.1198108 | / |
| 72 | Substrate specificity of the pseudouridine synthase RluD in Escherichia coli | 17937767 | 10.1111/j.1742-4658.2007.06101.x | / |
| 73 | Base pairing within the psi32,psi39-modified anticodon arm of Escherichia coli tRNA(Phe) | 17147349 | 10.1021/ja0659368 | / |
| 74 | Molecular dynamics simulations of human tRNA Lys,3 UUU: the role of modified bases in mRNA recognition | 17012271 | 10.1093/nar/gkl580 | / |
| 75 | Formation of the conserved pseudouridine at position 55 in archaeal tRNA | 16920741 | 10.1093/nar/gkl530 | / |
| 76 | Solution structure of psi32-modified anticodon stem-loop of Escherichia coli tRNAPhe | 16377777 | 10.1093/nar/gki1004 | / |
| 77 | Pseudouridine modification in Caenorhabditis elegans spliceosomal snRNAs: unique modifications are found in regions involved in snRNA-snRNA interactions | 16236171 | 10.1186/1471-2199-6-20 | / |
| 78 | Effects of nucleotide substitution and modification on the stability and structure of helix 69 from 28S rRNA | 16120833 | 10.1261/rna.2320605 | / |
| 79 | Pseudouridylation of yeast U2 snRNA is catalyzed by either an RNA-guided or RNA-independent mechanism | 15962000 | 10.1038/sj.emboj.7600718 | / |
| 80 | The pseudouridine synthase RluD is required for normal ribosome assembly and function in Escherichia coli | 15928344 | 10.1261/rna.2550105 | / |
| 81 | Number, position, and significance of the pseudouridines in the large subunit ribosomal RNA of Haloarcula marismortui and Deinococcus radiodurans | 15659360 | 10.1261/rna.7209905 | / |
| 82 | Human box H/ACA pseudouridylation guide RNA machinery | 15199136 | 10.1128/MCB.24.13.5797-5807.2004 | / |
| 83 | Crystal structures of the catalytic domains of pseudouridine synthases RluC and RluD from Escherichia coli | 15078091 | 10.1021/bi036079c | / |
| 84 | Crystal structure of the catalytic domain of RluD, the only rRNA pseudouridine synthase required for normal growth of Escherichia coli | 14730022 | 10.1261/rna.5187404 | / |
| 85 | A snoRNA that guides the two most conserved pseudouridine modifications within rRNA confers a growth advantage in yeast | 12810910 | 10.1261/rna.5240503 | / |
| 86 | Pseudouridines and pseudouridine synthases of the ribosome | 12762017 | 10.1101/sqb.2001.66.147 | / |
| 87 | Caenorhabditis elegans pseudouridine synthase 1 activity in vivo: tRNA is a substrate, but not U2 small nuclear RNA | 12597772 | 10.1042/BJ20021938 | / |
| 88 | Synthesis of helix 69 of Escherichia coli 23S rRNA containing its natural modified nucleosides, m(3)Psi and Psi | 12467398 | 10.1021/jo026364m | / |
| 89 | Lack of pseudouridine 38/39 in the anticodon arm of yeast cytoplasmic tRNA decreases in vivo recoding efficiency | 12058040 | 10.1074/jbc.M203456200 | / |
| 90 | Pseudouridylation of U(35) in the anticodon of Arabidopsis thaliana pre-tRNA(Tyr) depends on length rather than structure of an intron | 11955622 | 10.1016/s0167-4781(01)00355-4 | / |
| 91 | Role of editing in plant mitochondrial transfer RNAs | 11943456 | 10.1016/s0378-1119(01)00817-4 | / |
| 92 | Identification and site of action of the remaining four putative pseudouridine synthases in Escherichia coli | 11720289 | / | / |
| 93 | Genetic approaches to studying protein synthesis: effects of mutations at Psi516 and A535 in Escherichia coli 16S rRNA | 11694635 | 10.1093/jn/131.11.2994S | / |
| 94 | A second function for pseudouridine synthases: A point mutant of RluD unable to form pseudouridines 1911, 1915, and 1917 in Escherichia coli 23S ribosomal RNA restores normal growth to an RluD-minus strain | 11453071 | 10.1017/s1355838201000243 | / |
| 95 | Isolation and properties of Escherichia coli 23S-RNA pseudouridine 1911, 1915, 1917 synthase (RluD) | 11087118 | 10.1080/15216540050176566 | / |
| 96 | Unique structural and stabilizing roles for the individual pseudouridine residues in the 1920 region of Escherichia coli 23S rRNA | 10773075 | 10.1093/nar/28.10.2075 | / |
| 97 | Identification of the Saccharomyces cerevisiae RNA:pseudouridine synthase responsible for formation of psi(2819) in 21S mitochondrial ribosomal RNA | 10756195 | 10.1093/nar/28.9.1941 | / |
| 98 | The first determination of pseudouridine residues in 23S ribosomal RNA from hyperthermophilic Archaea Sulfolobus acidocaldarius | 10580099 | 10.1016/s0014-5793(99)01524-0 | / |
| 99 | Structural and functional roles of the N1- and N3-protons of psi at tRNA's position 39 | 10446245 | 10.1093/nar/27.17.3543 | / |
| 100 | Functional effect of deletion and mutation of the Escherichia coli ribosomal RNA and tRNA pseudouridine synthase RluA | 10383384 | 10.1074/jbc.274.27.18880 | / |
| 101 | The presence of pseudouridine in the anticodon alters the genetic code: a possible mechanism for assignment of the AAA lysine codon as asparagine in echinoderm mitochondria | 10076000 | 10.1093/nar/27.7.1683 | / |
| 102 | Identification and characterization of the tRNA:Psi 31-synthase (Pus6p) of Saccharomyces cerevisiae | 11406626 | 10.1074/jbc.M103131200 | / |
| 103 | Pseudouridine synthase 3 from mouse modifies the anticodon loop of tRNA. | 11027153 | 10.1021/bi001109m | / |
| 104 | Effect of modified nucleotides on Escherichia coli tRNAGlu structure and on its aminoacylation by glutamyl-tRNA synthetase. Predominant and distinct roles of the mnm5 and s2 modifications of U34. | 10583410 | 10.1046/j.1432-1327.1999.00965.x | / |
| 105 | Identification of two Escherichia coli pseudouridine synthases that show multisite specificity for 23S RNA | 9843401 | 10.1021/bi981002n | / |
| 106 | A pseudouridine synthase required for the formation of two universally conserved pseudouridines in ribosomal RNA is essential for normal growth of Escherichia coli | 9814761 | 10.1017/s1355838298981146 | / |
| 107 | Pseudouridine and ribothymidine formation in the tRNA-like domain of turnip yellow mosaic virus RNA | 9705510 | 10.1093/nar/26.17.3991 | / |
| 108 | The yeast tRNA:pseudouridine synthase Pus1p displays a multisite substrate specificity | 9671058 | 10.1017/s1355838298980396 | / |
| 109 | An RNA model system for investigation of pseudouridine stabilization of the codon-anticodon interaction in tRNALys, tRNAHis and tRNATyr | 9669557 | 10.1080/07391102.1998.10509006 | / |
| 110 | The rluC gene of Escherichia coli codes for a pseudouridine synthase that is solely responsible for synthesis of pseudouridine at positions 955, 2504, and 2580 in 23 S ribosomal RNA | 9660827 | 10.1074/jbc.273.29.18562 | / |
| 111 | The yeast gene YNL292w encodes a pseudouridine synthase (Pus4) catalyzing the formation of psi55 in both mitochondrial and cytoplasmic tRNAs | 9358157 | 10.1093/nar/25.22.4493 | / |
| 112 | Modification of human U4 RNA requires U6 RNA and multiple pseudouridine synthases | 9365261 | 10.1093/nar/25.23.4808 | / |
| 113 | Major identity determinants for enzymatic formation of ribothymidine and pseudouridine in the T psi-loop of yeast tRNAs | 9417931 | 10.1006/jmbi.1997.1417 | / |
| 114 | Mapping to nucleotide resolution of pseudouridine residues in large subunit ribosomal RNAs from representative eukaryotes, prokaryotes, archaebacteria, mitochondria and chloroplasts | 9047361 | 10.1006/jmbi.1996.0737 | / |
| 115 | The role of cysteine residues in the rearrangement of uridine to pseudouridine catalyzed by pseudouridine synthase I | 8999885 | 10.1074/jbc.272.3.1950 | / |
| 116 | Localization of modified nucleotides in Schizosaccharomyces pombe spliceosomal small nuclear RNAs: modified nucleotides are clustered in functionally important regions | 8809017 | / | / |
| 117 | Structural characterization of U*-1915 in domain IV from Escherichia coli 23S ribosomal RNA as 3-methylpseudouridine | 8604311 | 10.1093/nar/24.4.688 | / |
| 118 | A novel enzymatic pathway leading to 1-methylinosine modification in Haloferax volcanii tRNA | 7501451 | 10.1093/nar/23.21.4312 | / |
| 119 | The pseudouridine residues of ribosomal RNA | 8722007 | 10.1139/o95-099 | / |
| 120 | Mapping of the 13 pseudouridine residues in Saccharomyces cerevisiae small subunit ribosomal RNA to nucleotide resolution | 7545286 | 10.1093/nar/23.16.3290 | / |
| 121 | A dual-specificity pseudouridine synthase: an Escherichia coli synthase purified and cloned on the basis of its specificity for psi 746 in 23S RNA is also specific for psi 32 in tRNA(phe) | 7493321 | / | / |
| 122 | Implications of a functional large ribosomal RNA with only three modified nucleotides. | 7541254 | 10.1016/0300-9084(96)88101-6 | / |
| 123 | Clustering of pseudouridine residues around the peptidyltransferase center of yeast cytoplasmic and mitochondrial ribosomes | 7947756 | 10.1021/bi00249a036 | / |
| 124 | The single pseudouridine residue in Escherichia coli 16S RNA is located at position 516 | 7524026 | 10.1093/nar/22.18.3681 | / |
| 125 | Formation of pseudouridine in U5 small nuclear RNA | 8068680 | 10.1021/bi00200a025 | / |
| 126 | Four newly located pseudouridylate residues in Escherichia coli 23S ribosomal RNA are all at the peptidyltransferase center: analysis by the application of a new sequencing technique | 8373778 | 10.1021/bi00088a030 | / |
| 127 | 15N-labeled Escherichia coli tRNAfMet, tRNAGlu, tRNATyr, and tRNAPhe. Double resonance and two-dimensional NMR of N1-labeled pseudouridine | 3894360 | / | / |
| 128 | Conformational study of low molecular weight RNAs in solution by fluorescent probe | 6395094 | / | / |
| 129 | Nuclear Overhauser effect study of yeast tRNAVal 1: evidence for uridine-pseudouridine base pairing | 6761651 | 10.1093/nar/10.24.8297 | / |
| 130 | Epitranscriptome machinery in Trypanosomatids: New players on the table? | 33513291 | 10.1111/mmi.14688 | / |
| 131 | Halobacterium volcanii tRNAs. Identification of 41 tRNAs covering all amino acids, and the sequences of 33 class I tRNAs | 6746655 | / | / |
| 132 | Unconventional decoding of the AUA codon as methionine by mitochondrial tRNAMet with the anticodon f5CAU as revealed with a mitochondrial in vitro translation system | 19151083 | 10.1093/nar/gkp001 | / |

*Note:* “/” represents no data available.
