# Supplementary material for: Ψ-Atlas: An Integrated Atlas for Pseudouridine Epitranscriptome
Source: Genomics Proteomics Bioinformatics. 2026 Jan 23;24(2):qzag004. doi: 10.1093/gpbjnl/qzag004 (PMC13529368; doi:10.1093/gpbjnl/qzag004)
Supplement: Supplementary file 1 [file qzag004_supplementary_data.zip › Table S3.docx]

**Table S3 Data sources and bioinformatics workflow used tools in Ψ-Atlas**

| **Name** | **Links** | **Ref.** |
| --- | --- | --- |
| **Data sources** | | |
| Ensembl database | https://ensembl.org/ | [1] |
| NCBI | https://www.ncbi.nlm.nih.gov/ |  |
| Phytozome13 | https://phytozome-next.jgi.doe.gov/ | [2] |
| RMDisease V2.0 | RMDisease V2.0 | [3] |
| EPITOMY | https://epitomy.luddy.indianapolis.iu.edu/ |  |
| DirectRMDB | <http://www.rnamd.org/directRMDB/> | [4] |
| RMBase v3.0 | https://rna.sysu.edu.cn/rmbase3/ | [5] |
| PRMD | http://bioinformatics.sc.cn/PRMD | [6] |
| GWAS Atlas | <https://ngdc.cncb.ac.cn/gwas/> | [7] |
| PlantAPAdb | http://www.bmibig.cn/plantAPAdb/ | [8] |
| G4ATLAS | https://www.g4atlas.org/ | [9] |
| R-loopAtlas | http://bioinfor.kib.ac.cn/R-loopAtlas/l2/browse_obser.html | [10] |
| RASP | http://rasp.zhanglab.net | [11] |
| PsORF | [http://psorf.whu.edu.cn](http://psorf.whu.edu.cn/#/) | [12] |
| POSTAR3 | http://111.198.139.65/index.html | [13] |
| AtMAD | <http://119.3.41.228/atmad/index.php> | [14] |
| Rice-eQTL | http://riceqtl.ncpgr.cn/home/ |  |
| **Bioinformatics tools** | | |
| Gffread | <https://github.com/gpertea/gffread> | [15] |
| Bedtools | <https://github.com/arq5x/bedtools2> | [16] |
| JBrowse | https://jbrowse.org/jb2/ | [17] |
| NanoPsu | https://github.com/sihaohuanguc/Nanopore_psU | [18] |
| NanoSpa | https://github.com/sihaohuanguc/NanoSPA | [19] |
| TandemMod | https://github.com/yulab2021/TandemMod | [20] |
| Leftover | https://genome-store.ucsc.edu/ | [21] |

**References**

[1] Howe KL, Achuthan P, Allen J, Allen J, Alvarez-Jarreta J, Amode MR, et al. Ensembl 2021. Nucleic Acids Res 2021;49:D884–91.

[2] Goodstein DM, Shu S, Howson R, Neupane R, Hayes RD, Fazo J, et al. Phytozome: a comparative platform for green plant genomics. Nucleic Acids Res 2012;40:D1178–86.

[3] Song B, Wang X, Liang Z, Ma J, Huang D, Wang Y, et al. RMDisease V2.0: an updated database of genetic variants that affect RNA modifications with disease and trait implication. Nucleic Acids Res 2023;51:D1388–96.

[4] Zhang Y, Jiang J, Ma J, Wei Z, Wang Y, Song B, et al. DirectRMDB: a database of post-transcriptional RNA modifications unveiled from direct RNA sequencing technology. Nucleic Acids Res 2023;51:D106–16.

[5] Xuan J, Chen L, Chen Z, Pang J, Huang J, Lin J, et al. RMBase v3.0: decode the landscape, mechanisms and functions of RNA modifications. Nucleic Acids Res 2024;52:D273–84.

[6] Lang X, Yu C, Shen M, Gu L, Qian Q, Zhou D, et al. PRMD: an integrated database for plant RNA modifications. Nucleic Acids Res 2024;52:D1597–613.

[7] Liu X, Tian D, Li C, Tang B, Wang Z, Zhang R, et al. GWAS Atlas: an updated knowledgebase integrating more curated associations in plants and animals. Nucleic Acids Res 2023;51:D969–76.

[8] Zhu S, Ye W, Ye L, Fu H, Ye C, Xiao X, et al. PlantAPAdb: a comprehensive database for alternative polyadenylation sites in plants. Plant Physiol 2020;182:228–42.

[9] Yu H, Qi Y, Yang B, Yang X, Ding Y. G4Atlas: a comprehensive transcriptome-wide G-quadruplex database. Nucleic Acids Res 2023;51:D126–34.

[10] Li K, Wu Z, Zhou J, Xu W, Li L, Liu C, et al. R-loopAtlas: an integrated R-loop resource from 254 plant species sustained by a deep-learning-based tool. Mol Plant 2023;16:493–6.

[11] Li P, Zhou X, Xu K, Zhang QC. RASP: an atlas of transcriptome-wide RNA secondary structure probing data. Nucleic Acids Res 2021;49:D183–91.

[12] Chen Y, Li D, Fan W, Zheng X, Zhou Y, Ye H, et al. PsORF: a database of small ORFs in plants. Plant Biotechnol J 2020;18:2158–60.

[13] Zhao W, Zhang S, Zhu Y, Xi X, Bao P, Ma Z, et al. POSTAR3: an updated platform for exploring post-transcriptional regulation coordinated by RNA-binding proteins. Nucleic Acids Res 2022;50:D287–94.

[14] Lan Y, Sun R, Ouyang J, Ding W, Kim MJ, Wu J, et al. AtMAD: Arabidopsis thaliana multi-omics association database. Nucleic Acids Res 2021;49:D1445–51.

[15] Pertea G, Pertea M. GFF Utilities: GffRead and GffCompare. F1000Res 2020;9:ISCB Comm J-304.

[16] Quinlan AR. BEDTools: The Swiss-Army Tool for Genome Feature Analysis. Curr Protoc Bioinformatics 2014;47:11.12.1–34.

[17] Skinner ME, Uzilov AV, Stein LD, Mungall CJ, Holmes IH. JBrowse: a next-generation genome browser. Genome Res 2009;19:1630–8.

[18] Huang S, Zhang W, Katanski CD, Dersh D, Dai Q, Lolans K, et al. Interferon inducible pseudouridine modification in human mRNA by quantitative nanopore profiling. Genome Biol 2021;22:330.

[19] Huang S, Wylder AC, Pan T. Simultaneous nanopore profiling of mRNA m(6)A and pseudouridine reveals translation coordination. Nat Biotechnol 2024;42:1831–5.

[20] Wu Y, Shao W, Yan M, Wang Y, Xu P, Huang G, et al. Transfer learning enables identification of multiple types of RNA modifications using nanopore direct RNA sequencing. Nat Commun 2024;15:4049.

[21] Kent WJ, Sugnet CW, Furey TS, Roskin KM, Pringle TH, Zahler AM, et al. The human genome browser at UCSC. Genome Res 2002;12:996–1006.
