# Supplementary material for: Ψ-Atlas: An Integrated Atlas for Pseudouridine Epitranscriptome
Source: Genomics Proteomics Bioinformatics. 2026 Jan 23;24(2):qzag004. doi: 10.1093/gpbjnl/qzag004 (PMC13529368; doi:10.1093/gpbjnl/qzag004)
Supplement: Supplementary file 1 [file qzag004_supplementary_data.zip › Table S4.docx]

**Table S4 The results of PsiVar analysis**

| **Chr** | **Pos** | **Ref** | **Alt** | **TranscriptID** | **Strand** | **peak ID** | **MOTIF ref** | **MOTIF alt** | **Score ref** | **Score alt** | **gainorlost** |
| --- | --- | --- | --- | --- | --- | --- | --- | --- | --- | --- | --- |
| chr1 | 72495 | T | C | AT1G01160.1 | + | 1:72492 | NA | GTTCG | 0 | 1 | Gained |
| chr1 | 72495 | T | C | AT1G01160.2 | + | 1:72492 | NA | GTTCG | 0 | 1 | Gained |
| chr1 | 284600 | ACCTTT | AGCTTT,ATCTTT,AC | AT1G01790.1 | + | 1:290070 | NA | GTTCC | 0 | 0.939 | Gained |
| chr1 | 284600 | ACCTTT | AGCTTT,ATCTTT,AC | AT1G01790.2 | + | 1:290070 | NA | GTTCC | 0 | 0.939 | Gained |
| chr1 | 284686 | TC | TCGTCCCAA,TT | AT1G01790.2 | + | 1:290070 | NA | GTTCC | 0 | 0.939 | Gained |
| chr1 | 293388 | A | T,G | AT1G01800.1 | + | 1:294740 | NA | GTTCA | 0 | 0.999 | Gained |
| chr1 | 294906 | T | G | AT1G01800.1 | + | 1:294910 | GTTCG | NA | 1 | 0 | Lost |
| chr1 | 294908 | C | G | AT1G01800.1 | + | 1:294910 | GTTCG | NA | 1 | 0 | Lost |
| chr1 | 294909 | G | C | AT1G01800.1 | + | 1:294910 | GTTCG | GTTCC | 1 | 0.939 | Lost |
| chr1 | 293388 | A | T,G | AT1G01800.2 | + | 1:294740 | NA | GTTCA | 0 | 0.999 | Gained |
| chr1 | 294906 | T | G | AT1G01800.2 | + | 1:294910 | GTTCG | NA | 1 | 0 | Lost |
| chr1 | 294908 | C | G | AT1G01800.2 | + | 1:294910 | GTTCG | NA | 1 | 0 | Lost |
| chr1 | 294909 | G | C | AT1G01800.2 | + | 1:294910 | GTTCG | GTTCC | 1 | 0.939 | Lost |
| chr1 | 626779 | TA | T | AT1G02840.1 | + | 1:629703 | TGTAG | NA | 0.971 | 0 | Lost |
| chr1 | 629196 | TAGA | T | AT1G02840.2 | + | 1:629703 | TGTAG | NA | 0.971 | 0 | Lost |
| chr1 | 626779 | TA | T | AT1G02840.4 | + | 1:629703 | TGTAG | NA | 0.971 | 0 | Lost |
| chr1 | 629196 | TAGA | T | AT1G02840.4 | + | 1:629703 | TGTAG | NA | 0.971 | 0 | Lost |
| chr1 | 626779 | TA | T | AT1G02840.5 | + | 1:629703 | TGTAG | NA | 0.971 | 0 | Lost |
| chr1 | 629196 | TAGA | T | AT1G02840.5 | + | 1:629703 | TGTAG | NA | 0.971 | 0 | Lost |
| chr1 | 816360 | TCATTGGCTGAGC | T | AT1G03310.1 | + | 1:816433 | TGTAG | NA | 0.971 | 0 | Lost |
| chr1 | 816437 | A | G | AT1G03310.1 | + | 1:816433 | TGTAG | NA | 0.971 | 0 | Lost |
| chr1 | 816360 | TCATTGGCTGAGC | T | AT1G03310.2 | + | 1:816433 | TGTAG | NA | 0.971 | 0 | Lost |
| chr1 | 816437 | A | G | AT1G03310.2 | + | 1:816433 | TGTAG | NA | 0.971 | 0 | Lost |
| chr1 | 852334 | GGAAGAAGAAA | G | AT1G03440.1 | + | 1:852937 | GTTCA | NA | 0.999 | 0 | Lost |
| chr1 | 852661 | TGAAGAAGAA | TGAAGAA,T | AT1G03440.1 | + | 1:852937 | GTTCA | NA | 0.999 | 0 | Lost |
| chr1 | 852334 | GGAAGAAGAAA | G | AT1G03440.1 | + | 1:852938 | GTTCA | NA | 0.999 | 0 | Lost |
| chr1 | 852661 | TGAAGAAGAA | TGAAGAA,T | AT1G03440.1 | + | 1:852938 | GTTCA | NA | 0.999 | 0 | Lost |
| chr1 | 852334 | GGAAGAAGAAA | G | AT1G03440.1 | + | 1:853446 | GTTCA | NA | 0.999 | 0 | Lost |
| chr1 | 852353 | AGAC | A | AT1G03440.1 | + | 1:853446 | GTTCA | NA | 0.999 | 0 | Lost |
| chr1 | 852661 | TGAAGAAGAA | TGAAGAA,T | AT1G03440.1 | + | 1:853446 | GTTCA | NA | 0.999 | 0 | Lost |
| chr1 | 1051592 | GATTTTTGTCCT | G | AT1G04080.4 | + | 1:1051777 | TGTAG | NA | 0.971 | 0 | Lost |
| chr1 | 1051765 | G | A | AT1G04080.4 | + | 1:1051777 | TGTAG | NA | 0.971 | 0 | Lost |
| chr1 | 1099808 | G | C | AT1G04170.1 | + | 1:1099822 | GTTCG | NA | 1 | 0 | Lost |
| chr1 | 1099810 | T | A | AT1G04170.1 | + | 1:1099822 | GTTCG | NA | 1 | 0 | Lost |
| chr1 | 1099811 | C | A | AT1G04170.1 | + | 1:1099822 | GTTCG | NA | 1 | 0 | Lost |
| chr1 | 1099812 | G | A | AT1G04170.1 | + | 1:1099822 | GTTCG | GTTCA | 1 | 0.999 | Lost |
| chr1 | 1099808 | G | C | AT1G04170.2 | + | 1:1099822 | GTTCG | NA | 1 | 0 | Lost |
| chr1 | 1099810 | T | A | AT1G04170.2 | + | 1:1099822 | GTTCG | NA | 1 | 0 | Lost |
| chr1 | 1099811 | C | A | AT1G04170.2 | + | 1:1099822 | GTTCG | NA | 1 | 0 | Lost |
| chr1 | 1099812 | G | A | AT1G04170.2 | + | 1:1099822 | GTTCG | GTTCA | 1 | 0.999 | Lost |
| chr1 | 1232909 | C | T | AT1G04520.1 | + | 1:1232902 | NA | GTTCC | 0 | 0.939 | Gained |
| chr1 | 1233856 | TCTTTGAGG | T | AT1G04520.1 | + | 1:1233941 | GTTCA | NA | 0.999 | 0 | Lost |
| chr1 | 1293331 | A | G | AT1G04640.1 | + | 1:1293314 | NA | GTTCA | 0 | 0.999 | Gained |
