# Supplementary material for: Ψ-Atlas: An Integrated Atlas for Pseudouridine Epitranscriptome
Source: Genomics Proteomics Bioinformatics. 2026 Jan 23;24(2):qzag004. doi: 10.1093/gpbjnl/qzag004 (PMC13529368; doi:10.1093/gpbjnl/qzag004)
Supplement: Supplementary file 1 [file qzag004_supplementary_data.zip › Supplementary material captions.docx]

**Supplementary material**

**Table S1 Collection of Ψ modification sites in Ψ-Atlas**

**Table S2 Collection of Ψ modifications datasets resource**

**Table S3 Data sources and bioinformatics workflow used tools in Ψ-Atlas**

**Table S4 The results of PsiVar analysis**
